# Efficient remediation of antibiotic pollutants from the environment by innovative biochar: current updates and prospects

**DOI:** 10.1080/21655979.2022.2108564

**Published:** 2022-09-13

**Authors:** Ravi Katiyar, Chiu-Wen Chen, Reeta Rani Singhania, Mei-Ling Tsai, Ganesh D. Saratale, Ashok Pandey, Cheng-Di Dong, Anil Kumar Patel

**Affiliations:** a Institute of Maritime Science and Technology, National Kaohsiung University of Science and Technology, Kaohsiung City, Kaohsiung, 81157, Taiwan; b Department of Marine Environmental Engineering, National Kaohsiung University of Science and Technology, Kaohsiung City, Kaohsiung, 81157, Taiwan; c Sustainable Environment Research Center, National Kaohsiung University of Science and Technology, Kaohsiung City, 81157, Taiwan; d Centre for Energy and Environmental Sustainability, Lucknow 226 029, India; e Department of Seafood Science, National Kaohsiung University of Science and Technology, Kaohsiung city, Kaohsiung, 81157, Taiwan; f Department of Food Science and Biotechnology, Dongguk University-Seoul, Ilsandong-gu, Goyang-si 10326, South Korea; g Centre for Innovation and Translational Research, CSIR-Indian Institute of Toxicology Research, Lucknow, Uttar Pradesh, 226 001, India; h Sustainability Cluster, School of Engineering, University of Petroleum and Energy Studies, Dehradun 248 007, India; i Institute of Aquatic Science and Technology, National Kaohsiung University of Technology, Kaohsiung City, 81157, Taiwan

**Keywords:** Biochar, antibiotics, adsorption, biodegradation, bioremediation

## Abstract

The increased antibiotic consumption and their improper management led to serious antibiotic pollution and its exposure to the environment develops multidrug resistance in microbes against antibiotics. The entry rate of antibiotics to the environment is much higher than its exclusion; therefore, efficient removal is a high priority to reduce the harmful impact of antibiotics on human health and the environment. Recent developments in cost-effective and efficient biochar preparation are noticeable for their effective removal. Moreover, biochar engineering advancements enhanced biochar remediation performance several folds more than in its pristine forms. Biochar engineering provides several new interactions and bonding abilities with antibiotic pollutants to increase remediation efficiency. Especially heteroatoms-doping significantly increased catalysis of biochar. The main focus of this review is to underline the crucial role of biochar in the abatement of emerging antibiotic pollutants. A detailed analysis of both native and engineered biochar is provided in this article for antibiotic remediation. There has also been discussion of how biochar properties relate to feedstock, production conditions and manufacturing technologies, and engineering techniques. It is possible to produce biochar with different surface functionalities by varying the feedstock or by modifying the pristine biochar with different chemicals and preparing composites. Subsequently, the interaction of biochar with antibiotic pollutants was compared and reviewed. Depending on the surface functionalities of biochar, they offer different types of interactions e.g., π-π stacking, electrostatic, and H-bonding to adsorb on the biochar surface. This review demonstrates how biochar and related composites have optimized for maximum removal performance by regulating key parameters. Furthermore, future research directions and opportunities for biochar research are discussed.

## Graphical abstract

**Figure:**
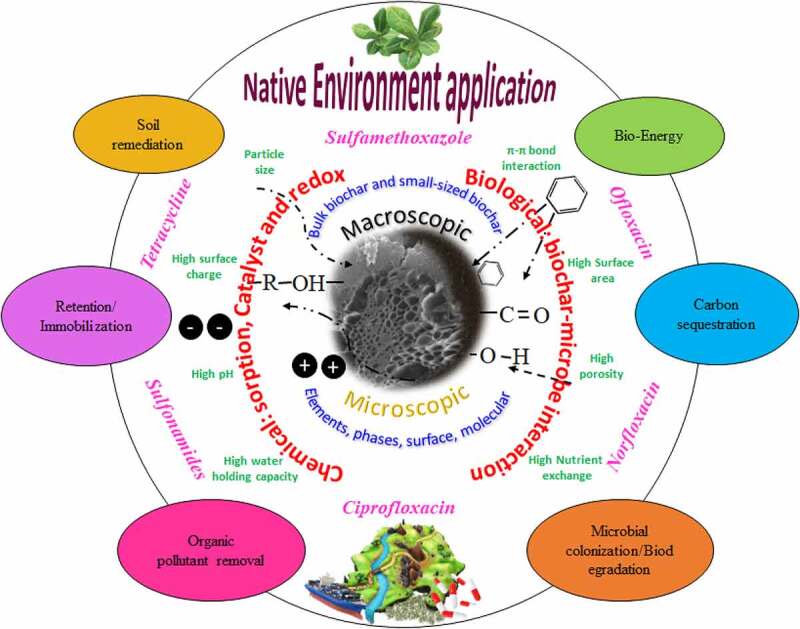

## 1. Introduction

Recent studies focused on the antibiotics used for several medical applications collectively covering natural, synthetic, and semi-synthetic molecules. Pharmaceutical products are greatly used for medicine and their residues are disposed of the environment. In research articles, the overall expenditure of antibiotics in livestock has been estimated at 63,151 tons in 2010 with the prediction of an increasing rate by 67% by 2030. On the basis of the global consumption of antibiotics 40.2 (95% uncertainty interval 37.2–43.7), billion defined daily doses population per day in 2018 with an increased rate of 46% [1, 2, 3]. The natural antibiotic types are derived from bacteria and fungi-based bioprocesses that are of great importance due to their high degradability. Antibiotics are complex molecules, each can have a different functional group and thus have a different chemical structure [4]. Antibiotics exhibit a dissociation constant (pKa) in the pH range of 1.5–9.5 depending on functional groups and thus they can be as neutral molecules (zwitterionic) or charged (negative or positive) molecules beyond that value [5]. Antibiotics like penicillin are easily degraded from the environment but the process of degradation is not the same for all other antibiotics such as Tetracycline, Sulfadiazine, Sulfonamide, Ciprofloxacin, Quinolones, etc. Such antibiotics are spread in wider forms as their uncontrolled and illegally disposed of in the environment along with their non-degradable property, they remain longer in the environment. The low rate of antibiotic degradation could maintain microbes under the minimal inhibitory concentrations (MICs) and must treat them below their MICs [6]. MIC is an important parameter that enables their persistence against degradation and maintains continuous production in that environment. Concentration below MIC can give rise to antibiotic-resistant bacteria. Antibiotics with less than 1000 D molecular weight indulged with beta-lactams, macrolides, quinolone, rifamycin, and tetracyclines dissolve very fast in water [7]. In an aqueous and soil environment, antibiotic shows many comminations to living beings, for example, toxic challenges, allergic responses, loss of immunity, and may also facilitate drug-resistant microbes’ evolution [8–10]; therefore, it is necessary to develop cost-effective and efficient technologies to remediate antibiotic effectively. Since wastewater treatment plants (WWTPs) are generally designed to simply remove and discreetly degradable organic pollutants in the mg/L range, they cannot fully degrade pharmaceuticals including antibiotics. Although the solubility, polarity, volatility, absorbability, biodegradability, and stability of antibiotics vary across a wide range. However, they can be active at incredibly low concentrations (ng/L µg/L). In this respect, biochar could be the most promising and cost-effective alternative method for their effective removals. It is well known that traditional biological wastewater treatment facilities are not able to remove the antibiotic residues effectively [11]. A variety of methods have been used to remove antibiotics from water, including bio-electrochemical systems, heterogeneous photocatalysis, oxidation processes, microbiological methods, and adsorption methods [12,13]. Despite being highly adsorptive, biochars are suitable for the mitigation of the effects of antibiotic residues in manure, which propagate when manure is added to the soil [14].

Biochar is basically a carbon-rich material produced during pyrolysis that is produced by thermochemically decomposing biomass at a range of temperature of about 300–900°C without oxygen. The physicochemical removal/degradation of a specific group of antibiotics by biochar was often studied, these studies were more focused on their removal efficiency but less emphasized the detailed mechanism such as adsorption, hydrolysis, volatilization, biodegradation, etc. Biochar derived from plant biomass has been considered an effective carbonaceous sorbent for organic and inorganic contaminants (including antibiotics) based on its attractive characteristics *e.g*., high porosity, aromaticity, multiple anionic functional groups, and large surface area [15–17]. Reduced aromaticity can cause the weaken π–π interaction between the catalyst and pollutant mechanism which is specially performed with an aqueous solution, Fe-N modification reduces the aromaticity of biochar and the lower H/C ratio represents the higher aromaticity of biochar [18]. Moreover, newly emerged engineered applications with enhanced surface functionality, additional interaction, and porosity make them more efficient [19]. The determination of the adsorption efficiency of various biochar was carried out which were derived from various feedstocks for antibiotic remediation [20]. Biochar was precisely termed depending on produced materials such as biosolids biochar (BDB), cattle manure biochar, spent coffee ground biochar (SPGB), etc [21]. These biochars were potentially used in varying doses ranging between 1 and 10 g L^−1^ for the removal of many antibiotics (*viz*. tetracycline, trimethoprim, clarithromycin, erythromycin, ampicillin, sulfamethoxazole, ofloxacin) from aqueous solutions under pH range between 5 to 11 [21]. Depending on the biochar properties before and after modification their removal efficiencies for these antibiotic removals were significantly varied.

Biochar received enormous research focus because of its cost-effectiveness and it is more attractive due to its excellent adsorption capacities for organic pollutants from aqueous solutions [22,23]. Larger particles of biochar (>2 mm) are preferred to recover and reuse for remediation applications many times. However, separating the powdered biochar from the aqueous solution is challenging hence inhibiting its adequate use in bioremediation applications [24]. In addition to the carbon element, porous structure, surface free radicals’ abundant functional groups, affect the corresponding action and function of any biochar [25]. New technologies like oxidation, coagulation, membrane filtrations, etc. are being developed in wastewater treatment plants (WWTPs) to remove antibiotics. These practices are more efficient but also increase the capital and operating costs to handle large volumes of wastewater [26]. A cost-effective solution is a prerequisite to concentrating them or binding them with any material that makes it easy and feasible for their treatment. Moreover, for plant-derived waste biomass, the common practice is composting or direct burning that is not a solution as it causes carbon emission [27].

This article discusses detailed research updates on the production and engineering of various biochar for the removal or degradation of antibiotic pollutants. Moreover, various antibiotic remediation mechanisms and remediation efficiencies have been summarized based on their physicochemical characteristics. It has also been discussed how biochar properties relate to feedstock, production conditions, technologies, and engineering methods and play role in antibiotic remediation. By varying the feedstock or modifying it with different methods (*e,g*., physical, chemicals, or biological) their surface functionalities (*e.g*., COO^−^, OH, M-O, etc.) and biodegradation ability are improved which applies to better remediation performance. Overall, this review is to discuss the cause and impacts of antibiotics on the environment. It also provides an overview of the performance of biochar and biochars plausibility for removal of antibiotic pollutants with major emphasis on regulating parameters besides the modification approaches. Furthermore, future research directions and opportunities for biochar research are discussed.

## 2. Biochar production route from biomass wastes

Biomass feedstock can be any type of residue from a crop, forest, seaweed, manure, etc. that can be used for biochar preparation as an alternative solution [28–30]. Worldwide, researchers are exploring renewable energy resources for feasible biochar production [31], and lignocellulosic biomass (LCB) is one of the renewable energy sources and most abundantly available resource which can be converted into desirable biochar. In addition to biochar production, LCB biomass has been reported for several other applications in recent years under bioprocess engineering [32–36]. During LCB biochar production three products are obtained: solid biochar, liquid bio-oil, and gaseous mix (CO_2_, CO, CH_4_, H_2,_ etc). Three product ratios are varied depending on biomass type and production condition and biochar is likely to be obtained more in a lower temperature range [16,37,38]. Energy extraction is often linked with the biochar production process in which biochar is a stable carbon-rich byproduct produced from the carbonization of biomass at different temperatures; however, other energy carriers were also obtained as the main products such as bio-oil and syngas. Biochar has a wide range of advanced environmental applications [39]. The above conversion process is basically carried out by biochemical conversion and thermochemical conversion. The thermochemical conversion technique is divided into combustion, gasification, and pyrolysis. A variety of methods have been developed to produce biochar by pyrolysis [37]. Pyrolysis is an effective, efficient, and sustainable process to generate biochar. It is a thermochemical process that occurs in the temperature range between 300°C and 1200°C [40,41]. The changes in the structure of the surface area, functional groups, and physicochemical properties of biochar are strongly related to a pyrolysis temperature. At a higher temperature, the surface area of biochar and porosity is increased because of the defilement of aliphatic alkyls and esters groups of the organic compounds, thereby eliminating the pore-blocking substances [16,17]. The high lignin-containing lignocellulosic biomass produces macroporous structures in the resulting biochar, while high cellulosic biomass primarily produces biochar with microporous structures [42].

The effective removal of antibiotics is accomplished by native biochar mainly produced at a middle-temperature range of 450–650°C [43, 44; 45, 46]. However, temperature selection goes a little higher side in the case of biochar modification in the majority of studies ranging from 350°C to 800°C [44,47–50]. According to a previous study, the biochar property for antibiotic pollutants removal greatly depends on the production conditions as well as biomass types, and the resultant surface property plays the role of specific interactions for pollutant adsorption [51]. In a previous study, the overall adsorption process was based on the method selection for converting the biomass into biochar, and the adsorption process onto the biochar surface was mainly carried out by π–π interaction between temperatures 350–650°C [52]. Chemical bonds of biomass are usually broken down at this temperature and started rearranging these bonds before forming new functional groups such as anhydride, lactol, pyridine, pyridine, quinine, chromene, etc [53]. The long carbon chain breakdowns into several small organic/inorganic molecules and the process yields the final product in three phases: gas phase: syngas, liquid phase: bio-oil, water, and tar, and solid-phase carbonaceous biochar [54]. Table 1 summarizes the types of feedstocks used for biochar production and their successive application for antibiotic removal.

**Table 1.** Application of various pristine biochar for antibiotic removal, adsorption mechanisms and their efficiencies.

Feedstocks	Pyrolysis Temp. (^o^C)	Targeted Antibiotic	Max. Adsorption [mg/g)	Sorption Mechanism	References
Municipal sewage sludge	800	Tetracycline	100	Graphitic C and N species were proved to be the catalytic sites	[55]
Pine Sawdust	650	Sulfamethoxazole	13.83	Hydrophobic interaction	[56]
Bamboo	550	sulfamethoxazole		π – π electron donor–acceptor interactions	[57]
Swine Manure	700	Tetracycline	109.5	H-bonding, π- π electron donor––acceptor interaction	[44]
Rice straw	700	Tetracycline	132.7	H-bonding, π- π electron donor–acceptor interaction	[58]
Peanut shells	450	Doxycycline hydrochloride	52.37	Strong complexation, electrostatic interactions	[65]
Eucalyptus sawdust	500	Dimetridazole	200.00	Physisorption, chemisorption	[59]
Eucalyptus sawdust	500	Metronidazole	167.50	Physisorption, chemisorption	[59,60]
Chitosan/biochar	450	Ciprofloxacin	80.29	π- π electron donor-acceptor interaction, H- bonding	[43]
Coconut Shells	500	Tetracycline	94.2	Hydrogen bonding, π – π EDA	[61]
Fe/Zn	600	Tetracycline	102.00	Electrostatic interaction, π – π electron donor––acceptor interaction	[62]
Wasted Sludge	500	Tetracycline adsorption	183.01	Electrostatic attraction, π – π stacking, pore filling, silicate bonding, chelating & ion exchange	[46]
Rice Straw	700	Tetracycline	153.7	Adsorption	[58]
Rice husk	500	Tetracycline	55.9	Calcination of Co[NO_3_]_2_ treated BCs	[63]
Camphor leaves	650	Ciprofloxacin	449.4	Intense π-π stacking interaction, electrostatic interaction & cation exchange interaction	[45]
Bamboo	600	Ciprofloxacin and Norfloxacin	245.6/ 293.2	Hydrophobic surface interactions, π-π electron donor––acceptor interaction, and electrostatic attraction	[64]

## 3. Mechanism and effect of key parameters for maximum biochar removal performance

Regardless of the intrinsic potential of biochar or modified biochar to interact with antibiotic pollutants, the study reveals the key regulating factors, especially pH is crucial to increasing removal potential several folds. For example, a previous study tested the tetracycline removal potential of H_3_PO_4_ modified two biochar derived from manure and rice straw. Both biochars have shown removal efficiencies ranging from 141 to 154 mg/g at pH 5 mainly involving chemisorptions including H-bonding and π-π electron donor–acceptor interactions. Moreover, these adsorption efficiencies were significantly strengthened by electrostatic attraction between biochars and tetracycline (TC] by increasing the pH from 5.0 to 9.0 which can largely explain the enhanced removal capacity of TC up to 365.4 and 552.0 mg/g, respectively, for manure and rice straw-derived biochars [44]. This study shows that based on parameter regulations the removal efficiency of biochar could increase 2.6–3.6-fold than their potential at pH 5 [44]. Another study also addressed that TC removal was greatly affected by pH regulation; however, the efficiency was increased by lowering the pH. This study emphasized that due to the amphoteric structure of TC its removal is greatly dependent on the pH of the solution. This work used sludge-derived biochar modified by chitosan and Fe/S (BCFe/S) for TC removal. The highest adsorption of BCFe/S obtained was 75.36 mg/g at pH 9, and 180 mg/g at pH 5 for biochar-Fe/S_4_, respectively [46]. For the adsorption process by Biochar: π-π stacking, electrostatic attraction, pore filling, silicate bonding, and H-bonding were the interactions for TC removal. Besides these mechanisms, BCFe/S-4 also exhibited chelating and ion-exchange mechanisms for TC removals [46].

In other previous studies on adsorption including biochar with antibiotics especially TC has several ionizable groups that enable it to be cationic (TCH^3+^) when solution pH is reduced to 3.3, zwitterionic (TCH^2^), or partly anionic (TCH^−^, TC^2−^) at pH 3.3 to 9.7, and fully anionic (TC^2−^) at pH over 9.7. Moreover, the solution pH also affects the biochar surface charge [16,65]. Due to the heterogeneous effect of pH on both pollutants and biochar, a point of zero charge (pH_pzc_) was announced. The pH_pzc_ of biochar and BCFe/S-4 were 8.53 and 5.16 respectively. Therefore, according to the pH value, electrostatic attraction or repulsion works hence the removal efficiency greatly fluctuates along with the pH range [46].

The adsorption capacity of various biochar is greatly varied with varying antibiotics group [20,21]. These biochars were potentially used in varying doses ranging between 1–10 g L-1 for the removal of many antibiotics (tetracycline, trimethoprim, erythromycin, clarithromycin, ampicillin, ofloxacin, sulfamethoxazole) from aqueous solutions. In some studies, pH was the major regulating factor for attractive removal efficiency which was examined in the range of 5 to 11 [21]. In a batch process, 100 µg L-1 of the initial concentration of several antibiotics was efficiently removed. As measured by batch sorption experiments, all biochars applied at a low dose were capable of removing more than 70% and even 100% of TET, ERY, and CLA, whereas manure-derived biochar also removed AMP. By applying biochars at a dosage of 10 g L-1, efficient adsorption was achieved, resulting in fast (within 5 mins incubation) and accomplishing the removal of ERY, TET, CLA, and >85% removal of TMP and AMP. Despite this, biochars studied did not remove SMX and OFL [21]. Biochar exhibited a higher capacity of adsorption due to the surface complexation, H-bonding, and pore-filling effects in the overall mechanism, which usually results in a quick removal rate and is supported with best-fitted isotherm models [21]. The effect of biochar dosing is also exhibited by [66]. Biochar could effectively absorb ciprofloxacin, oxytetracycline, doxycycline hydrochloride, tetracycline hydrochloride, fluoroquinolones antibiotics. With an increase in biochar dosage up to 1.2 g L-1, the effectiveness of removing the three antibiotics also increased [66].

The adsorption of seven antibiotics from aqueous solutions has been previously studied, and the application of carbon materials (graphene and biochar) showed promising adsorption, with a maximum removal efficiency of 100% [67]. The observations made in this study centered largely on the π-π stackings between aromatic rings of antibiotics and both carbon materials. By applying the density functional theory (DFT), the adsorption energy also markedly increased with an increase in the number of π rings, highlighting the role of π-π stackings in the adsorption [67]. According to this study, antibiotic pollutants with more aromatic rings containing biochar (produced at higher temperature >800) or graphene should be used for easy removal of antibiotics from the environmental samples.

The effective removal of SMX and SPY antibiotics are attained by anaerobically digested bagasse-derived biochar produced at 600°C in which pH was the most regulating factor in achieving maximum removal efficiency [68]. The removal efficiency of SMX and SPY from aqueous media, respectively, achieved 54.38 and 8.60 mg/g. The dominant interaction reported was π-π stacking and the adsorption decreased with increasing solution pH [68]. Table 1 summarizes the removal efficiency of native biochar for various antibiotics and their sorption mechanisms.

**Figure 1.**
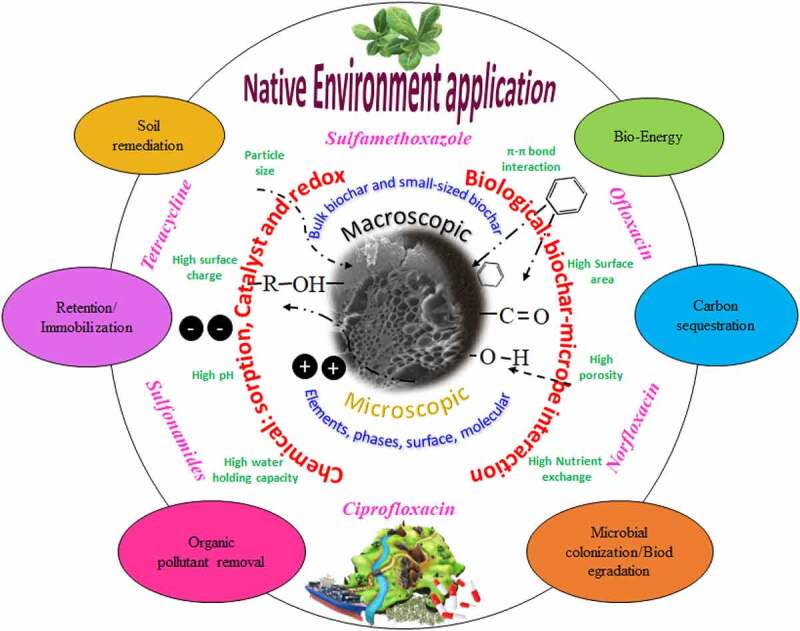
Schematic of pristine/modified biochar morphology, properties, and interactions for the removal/degradation of various antibiotics.

The effect of pyrolysis temperature is well understood on the resulting biochar surface properties. Low pyrolysis temperature results in less removal of biomass elements to form fewer pores, and more surface functional groups for ionic or polar interactions, however, porosity increases with increasing pyrolysis temperature and the resulting biochar are more aromatic and hydrophobic for non-polar interactions [16]. High porous biochar exhibits a higher adsorption capacity for antibiotics and other organic pollutants [69]. Biochar prepared at extremely higher pyrolysis temperature has high alkalinity and less favorable properties that can offer many interactions and better removal efficiency [70]. Biochar obtained at high pyrolysis temperature was reported to exhibit high removal capacity for tetracycline hydrochloride, doxycycline hydrochloride, and ciprofloxacin [71,72]. The effect of reaction temperature during pollutant interaction or adsorption mechanism is not well covered. Studies found that pollutant adsorption on biochar is an endothermic process and the adsorption rate increases with increasing reaction temperature [73]. Other studies also reported the effect of temperature (15–50°C) on food waste, manure, agro- and forestry waste-derived biochar and their adsorption for organic and inorganic pollutants which was spontaneous and endothermic in nature. Increasing reaction temperature favorably enhanced the adsorption process of both pollutants on the biochar surface [74]. The main mechanism of temperature on adsorption was reported due to an increase in the diffusion rate of pollutants with the increasing reaction temperature [75]. Figure 1 is a schematic of pristine/modified biochar morphology, properties, and interactions for the removal/degradation of various antibiotics.

## 4. Biochar modification strategies

Pyrolytic production and modification strategies of biochar is a remarkable substance as having the best adsorption qualities, biochar has compliance to adapt modification and during the modification procedure, it improves with one or more aspects such as surface area, pore-volume, aromaticity, more O-containing functional groups, ion-exchange property etc [76]. These modifications enable specific attachment to organic pollutants using additional covalent bonding, H-bonding, and electron––donor––acceptor of EDA bonding [76,77,]. There has been an intense focus on developing efficient ways to alter biochar to improve its performance several times over what it is currently doing. Based on the past studies, the most common methods of biochar modification are physical, biological, and chemical, among them the chemical method is most popular and thus greatly exploited.

The physical method (Gas, steam activation, pressure, electrochemical, UV, ultrasound, plasma, heat treatment, etc.) mainly improves the physicochemical properties of biochar, for example, surface area, pore-volume, pH, polarity, aromaticity, ash content, etc., [78,79]. Specifically, steam activation was found to increase the biochar hydrophilic property via improving the porous structure and acidic-functional groups whereas heat treatment enhanced the hydrophobic property of biochar by increasing basic-functional groups [80–82]. Biological modification is often involved in increasing the microbial or enzymatic activities on biochar surface and plays a crucial role in antibiotic degradation [83,84]. Doping of other biological materials prior to preparing biochar such as polysaccharides from animal or plant sources was also encouraging for improved biochar properties under biological modification [85,86]. Microorganisms enhance antibiotic remediation in both ways via enzymatic action as well as through electron exchange [19].

**Figure 2.**
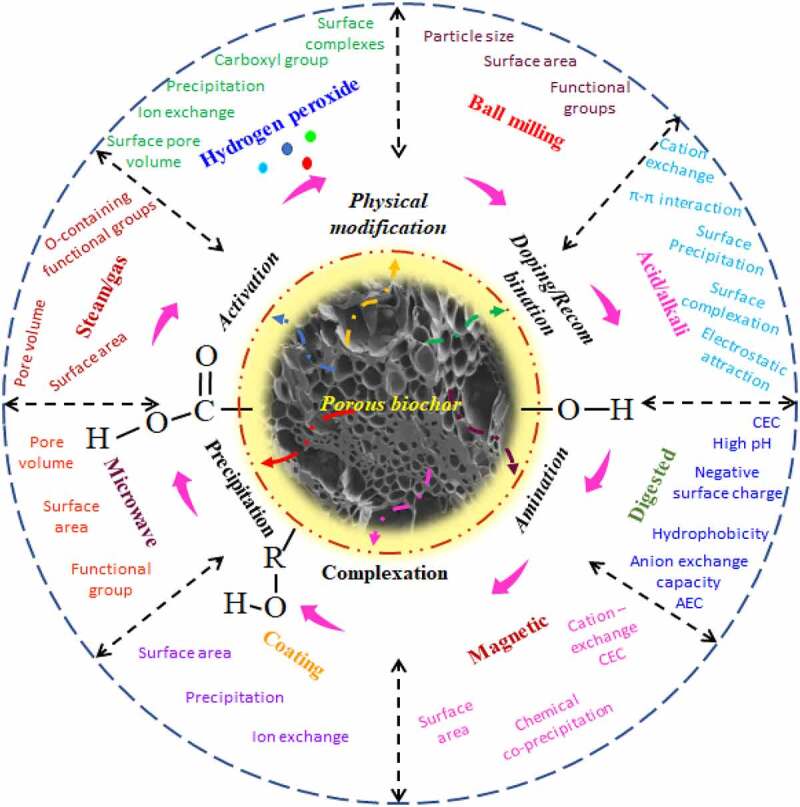
Representation of various modification methods on biochar surface to enhance the adsorption properties for antibiotic pollutants.

The chemical-based modification methods are carried out uniquely or in combination with the physical methods. It may apply acid, metal salts and oxides, and alkali in the majority of studies [87]. The acidic modification removes the minerals from carbon material and improves the acidic property and/or hydrophobicity of biochar [88]. Alkaline treatment of biochar produces a positive surface charge [80,81]. As compared to the physical method, chemical methods are more operative at enhancing the surface functionality of engineered biochar or alkali-modified biochar offers the maximum surface functionality [89]. On the other hand, the acidic modification enhances the oxygenated functional group’s counts on biochar surfaces [88,89]. The efficiency of the removal achieved using these methods differs based on the feedstock type and modification operating conditions [85,89–91]. The selection of an appropriate modification method based on native biochar characteristics is an important step, as each modification method must be complementary to enhance mechanism and adsorption performance. Antibiotic removal performance is greatly improved after various modification methods as summarized in Table 2. Figure 2 is the representation of various modification methods on biochar surfaces to enhance the adsorption properties of antibiotic pollutants. This modified biochar finds better bioremediation prospects than their pristine forms.

**Table 2.** Adsorption of antibiotics through modified biochar.

Feedstock	Modification Method	Biochar used (g/L)	Antibiotics	Pyrolysis Temp. (°C), time (h); N_2_ flow; heating rate (L min^−1^)	Removal efficiency [%)	References
Tea residue powder	Fe-BCK0.5-VB6	20	TC	700°C, 2 h, 10°C-min^−1^	90.89	[92]
Cassava	KOH	0.1	OTC	500°C	65.5–96.2	[47]
Date Palm Leave	Vit. B6 alginate	02	TC	500°C, 1 h, 100 ml/min	91–98.3	[93]
Waste Tea Residue	Fe_3_O_4_@T-BC	0.5	TC	500°C, 2 h	99.86	[94]
Raw Bamboo	Ball milling	10	SNM	300, 450 and 600°C, 1.5 h,	80	[95]
Hydrochar	Fe_2_O_3_	20	TC	300–700°C, 2 h, 1 L min^−1^	20	[12]
Date palm waste	Zeolite	5.6	CTC	600°C	30.42	[50]
Swine Manure	H_3_PO_4_	0.2	TC	700°C	60.9	[44]
Rice straw	H_3_PO_4_	0.2	TC	700°C	92	[58]
Shredded cotton stalks	H_2_O	–	SMX	350°C	68	[51]
Grapefruit peel	GPCB-20	9–10	TC	600°C, 1 h, 5°C-min^−1^	37.92	[55]
Vinasse	Fe/Mn	–	PEF	800°C		[48]
Date palm waste	Zeolite	–	CTC	600°C	30.42	[92]
Bagasse	Ball milled	10	SMX	300, 450 and 600°C, 1.5 h,	33.4–83.3	[47]
Vinasse	Fe/Mn	–	PEF	800°C		[48]
hickory chips	Ball milled -BB	10	SPY	300, 450 and 600°C, 1.5 h,	39.8–89.6	[47]
Camphor leaves	ZnO nanoparticle	0.5	CIP	650°C, 2 h	>75	[45]
Shredded cotton stalks	H_2_O	–	SMX	350°C	49	[51]
Bermuda grass	IA-BCs, π π EDA	20	SMX	800°C, 2 h, 2 L/min	62–64	[49]
Date palm waste	Zeolite	–	CTC	600°C	30.42	[92]
Sawdust	Co/Fe	–	CFT	500°C	99.23	[96]

Tetracycline (TC]; Sulfonamides (SNM); Sulfamethoxazole-SMX; Sulfapyridine (SPY); Iron FeCl_3_; activated biochar (IA-BCs); Tetracycline-TC, Trimethoprim-TMP, Erythromycin-ERY, Clarithromycin-CLA, Ampicillin-AMP, Ofloxacin-OFL, Sulfamethoxazole-SMX; Chlortetracycline-CTC; Ciprofloxacin-CIP; Tylosin-Tyl; Oxytetracycline -OTC; Norfloxacin -NOR; Levofloxacin-LEV; Doxycycline hydrochloride-DOX; pefloxacin (PEF)

## 4. Land application of biochar for antibiotic removal

Biochar is a promising antibiotic adsorbent not only in an aqueous environment for treating wastewater but also found effective for treating antibiotic polluted soil [16,97,98]. For further enhancement of the physical and chemical properties of biochar, several engineering methods were checked to improve the land application such as secondary carbonization, physical, chemical, and biological activation, and functional group doping treatments [19,89]. A high pyrolytic temperature increased the adsorption capacity of biochar for antibiotic residues [69]. Increasing pyrolysis temperature and biochar dosage improved the removal of doxycycline hydrochloride, tetracycline hydrochloride, and ciprofloxacin [71,72]. In a previous study, the best antibiotic adsorption was determined with plant biomass pyrolyzed at 700°C [66]. In order to adequately bind antibiotics, the properties of produced biochar are key factors, such as its sorption parameters, hydrophilicity, aromaticity, and O-containing surface functional groups [72]. In addition to antibiotic effects, biochar application also reduces the impact, availability, and bioavailability of pesticide, heavy metal, and antibiotic resistance genes in soil microbes [44].

For soil amelioration, pyrolyzed biochar can bind and degrade antibiotic pollutants in moist environments and also holds the moisture and nutrients that help to increase the crop yield [26,99]. Several studies demonstrated the biochar’s potential to block/reduce the negative effect of antibiotic residues in the production and quality of numerous crops. The role of biochar is promising in the bioavailability or transmission reduction of antibiotic pollutants to the soil microbes and food supply chain [44]. The presence of antibiotic pollutants is not limited to the wastewater, groundwater, and surface water but is also detected in the liquid manure, soil, and plant [72]. A major route for antibiotics to enter agricultural soils is through animal manure. Antibiotics can then be transported to other environmental compartments, including other human food-chain compartments [14]. Among various dissipation pathways for antibiotics in manure, adsorption is the dominant mechanism that governs its fate, transmission, and reactivity in the environmental samples and their effective removals [72,100]. Antibiotic remediation is often linked with the co-removal of heavy metals in many studies [49,101,102]. One latest study combined the waste-fungus-chaff-biochar (WFCB) and *Herbaspirillum huttiense* to bind copper and zinc before degrading both antibiotics enrofloxacin (ENR) and oxytetracycline (OTC). In the study, otcome showed that the combined material could immobilize Cu and Zn well (85.5 and 64.4%, respectively), and remove OTC 41.9% and ENR 40.7% [102].

Moreover, by developing antibiotic resistance genes (ARGs), microbes are becoming super antibiotic-resistant. It was reported last year that the persistence and dissemination of ARGs in soil bacteria posed one of the great threats to food security and public health [49,103]. Pathogens that are resistant to antibiotics are an emerging concern around the world and are considered a type of emerging contamination. There has been evidence that biochar blending can reduce the relative abundance of several subtypes of ARGs [104] because they can affect their dissemination and fate in the environment [105]. Several recent studies have investigated the use of biochar to alleviate ARG pollution in soil [105,106]. Several studies have notified that biochar can reduce ARG pollution in the soil to a certain extent, although not all biochar consistently did so. A recent study reported that 0.5% (w/w) rice straw biochar effectively reduces the abundance of 131 ARGs in non-planted soil, however, less effective in planted soil with *Brassica chinensis* L [44]. The biochar blending with soil effectively inhibited the ARGs conjugation frequency (gene transfer) between bacteria [105,107] affirming that ARGs replication was greatly inhibited via biochar interaction.

It was found that wheat straw biochar enhanced the relative abundance of tet and sul genes in the rhizosphere [101]. This suggests that biochar may not be as effective as previously thought in remediating ARG pollution. Specifically, this uncertainty relates to biochar properties that derive from feedstock and pyrolytic conditions, as they may affect the conjugative transfer of ARGs between bacteria [Liu et al., 108]. In addition, the amount of heavy metals and antibiotics in the soil determines the evolution of ARGs in the soil [49,101]. *Rhodanobacter, Brevundimonas, and Proteobacteria* were the hosts of ARGs [102]. One of the more intriguing aspects of research on biochars is the development of biochars with excellent efficiency in adsorbing heavy metals and antibiotics to protect soil from ARG pollution [49].

Biochar also works as a redox agent due to abundant bioavailable electrons in it and once it interacts with microbes can carry out biotic and abiotic transformations [19]. Microbial immobilized biochar effectively biodegrades organic pollutants as compared to the basic removal of pollutants via adsorption [109]. Moisture content and aeration were the most affecting or limiting factors to accelerating the degradation of organic contaminants by microbially immobilized biochar. Biochar mainly acts as a promising moderator of bioavailable electrons for organic pollutants degradation by microbes immobilized in biochar [107,110].

## 5. Microbial role in biochar mediated antibiotic remediations

To improve the antibiotic remediation efficiency further, some studies also applied microbial treatment on biochar surfaces [19,111]. Microbes are elegant in biodegrading antibiotics and other organic pollutants [112; 19]. Soil is the most suitable habitat for microorganisms. Thus, TC-polluted soil could be the most suitable source for isolating TC-degraders, as they may have promising bio-geo-chemical pathways [113]. Recent studies have closely examined the effect of antibiotics degrading microbes immobilized in biochar pores before their application in soil-plant systems to degrade antibiotic pollutants [111,114]. Microbes immobilized biochar is more efficient for antibiotic degradation rather than removal. A 10% (w/v) *Herbaspirillum huttiense* (Gram-negative bacteria) immobilized biochar exhibited maximum degradation efficiency of ENR with 3 wt% biochar application at 35°C [102]. Colonization of unidentified and favorable microbial communities can be easily identified in biochar communities using advanced technology [114]. Degradation studies still require advanced technologies for easy and precise determination of antibiotics as well as their metabolites in environmental samples [111]. Tetracyclines have low pKa and short half-life thus it is showing poor stability towards abiotic degradation. ENR however had a longer half-life but good adsorption affinity, thereby both antibiotics could be effectively reduced in soil by biochar +HH1 fluid applications [102]. For antibiotic degradation mainly two routes are referred to, biodegradable route and non-biodegradable routes, in which latter refers to several processes such as Ozonation, Photolysis, Fenton process, and advanced oxidation process [3]. Microbial degradation is different than non-biodegradable routes and is able to initiate antibiotic degradation by opening their loop structure or cleaving the enclosed functional groups involved in antibiosis.

Moreover, biochar was also effective in ARGs transmission reduction. *Rhodanobacter, Brevundimonas*, and *Proteobacteria* were the hosts of ARGs [102]. Total Phosphorus and pH greatly affected the antibiotic degradation by these microbes dwelling in biochar pores [102]. The abundance of *Proteobacteria, Rhodanobacter*, and *Brevundimonas* as potential hosts of ARGs altered due to biochar presence. Total phosphorus and pH were the factors driving the veterinary antibiotic degrading microorganisms and potential hosts of ARGs. It has also been demonstrated that bacteria have tolerance for antibiotics, as well as co-tolerance for heavy metals such as Cu, demonstrating the emerging potential of bacteria to utilize such compounds for ultimate antibiotic degradation [22,115]. A recent study employed their own isolates *Raoultella* sp. and *Pandoraea* sp. was capable of degrading 81.72% TC within 12 days of treatment [116]. The microbial community analysis of treated soil for TC degradation exhibited the abundance of four predominant phyla, *Bacteroidetes, Acidobacteria, Proteobacteria*, and *Chloroflexi* [111].

Biodegradation pathways have been described by several studies recently on TC. Based on eight metabolites, three putative TC degradation pathways by *Klebsiella* sp. are proposed: 1. reduction of an -OH group on C-3 then subsequent dehydration at C-12-a and C-6; 2. demethylation on C-4; 3. oxidation on C-5 and removal of -C = O on C-1 [117]. Other reports indicate that *Klebsiella* sp. (strain SQY5) biodegrades TC by removing the -CH_3_ functional group. Once the TC hydrolysis opened the ring, the -C = O group was removed, and later on removing the -NH_2_ group which led to the subsequent removal of two-CH_3_ and three-OH groups. A new putative degradation mechanism was proposed based on the authors’ identification of nine degradation products [118]. According to 119, *Stenotrophomonas maltophilia* DT1 demethylates TC at C-4, successively -C = O and -NH_2_ group removal. During TC degradation, when six biotransformation products were identified. There is a general consensus among the studies that TC biodegradation removes -CH_3_, -C = O, and -NH_2_ groups from the parent compound [111].

Biodegradation of antibiotics is reported to be more effective in the aqueous phase than that of sludge. AD is normally processed in a sludge dominating system in which the absolute quantity of antibiotic pollutants is more likely to be greater than the aqueous phase [120,121]. In the sludge system, antibiotic degradation is carried out sequentially: quick sludge sorption and desorption followed by degradation. The majority of studies found that AD is moderately effective up to 40–77% for antibiotic pollutants degradation depending on initial concentration. Both biosorption and biodegradation are key mechanisms for antibiotic remediation in AD processes. Increasing antibiotic accumulation is challenging to AD microbes for their effective removal in AD operation as well as biogas production. Biochar’s role can be pioneering to reduce the direct stress of antibiotics on AD microorganisms [122,123]. Biochar has been introduced recently as a conductive mediator into the AD system for improved organic degradation and methane production [122,123]. Especially engineered biochar with improved ability to increase the conductivity of mixed culture system to promote the methanogenesis is well covered using direct interspecies electron transfer (DIET) [124,125]. Biochar application in AD not only improved the biogas yield but also reinforced the microbial degradation of antibiotic pollutants via co-metabolism or electron exchange mechanism [122,123].

## 6. Factors affecting microbial degradation of antibiotics

To optimize the reaction conditions for effective TC biodegradation by various bacteria, researchers have regulated pH, Initial TC concentration, growth, and metabolic parameters. Some parameters are very crucial and affect the microbial degradation performance greatly. The pH is one of the most influencing factors for microbial degradation. A previous study focused on Stenotrophomonas maltophilia DT1 performance for TC biodegradation at different pH conditions [119]. With the initial pH of 10, the maximum TC hydrolysis was achieved; with an increase in pH, the hydrolysis rate was increased. The maximum TC biodegradation was obtained at pH 9 whereas microbial growth and activity were delayed when pH was 6 during the reaction initiation and exhibited 3 days of lag phase.

The initial concentration of antibiotics is also affecting the biodegradation rate significantly. TC antibiotics are susceptible to biodegradation based on their initial concentration, which determines the rate of biodegradation by microbes. During tests from 10 to 100 mg/L, Klebsiella sp. SQY5 degradation ratio tended to increase. The maximum degradation ratio reached 89.66% at 80 mg/L of initial TC concentration. However, the degradation ratio decreased later, it is motivating that strain SQY5 could use TC as an energy and carbon source, thus allowing it to survive a selection pressure of 100 mg/L of TC with a lesser degradation ratio [118]. The bacterium Stenotrophomonas maltophilia DT1 also exhibited the best fit of the Michaelis–Menten model for biodegradation kinetic of TC with respect to its initial concentration [119]. The degradation rate was increased up to 75 mg/L of the initial concentration of TC and thereafter decreased. Klebsiella pneumonia also shows up to 90% of TC degradation efficiency within 36 h treatment with the initial concentration of 200 mg/L under optimized conditions [117].

The effect of temperature on microbial degradation of antibiotics is inadequately reported. As per one previous report, the TC degradation rate was obtained at maximum in swine manure when the system temperature was 55 oC [126]. The study suggests that antibiotic degradation was improved during composting when thermal degradation and microbial degradation both work synergistically [127]. During the OTC degradation study, it was noted that maximum degradation of 65% was obtained after 100 h reaction at 40 oC [128], another study also reported that temperature and pH are the major affecting factor of OTC degradation [129]. Overall, the study suggests that the temperature range 40–55 was found to be suitable for antibiotic degradation depending on the mesophilic or thermophilic range of the employed microbial strain.

## 7. Challenges and prospects of organic pollutants removal by biochar-based adsorbents

From several recent studies, the majority of them mentioned their wider applications in bioremediation however limited studies have covered their negative and uneconomical perspectives. The researcher’s concerns should also be discussed clearly about their adverse effects on the environment. If any substance has certain toxic content and if it has not been disposed of appropriately. It may reach the surrounding with their potentially harmful effects, especially when used in higher quantities such as for soil amelioration. Biochar also has some harmful effects and it may be intensified when prepared at extremely higher pyrolysis temperature [70]. Such biochar may release some lethal polycyclic aromatic hydrocarbons (PAHs) and noxious heavy metals after soil amendment [70]. The long-term stability of biochar, based on its carbon structure has been considered one of the prime properties when it is applied in any method. Though it is reported that wood residue-derived and grass biomass-derived biochar usually are a sink of organic compounds like polycyclic aromatic hydrocarbons (PAHs), It contains two or more fused aromatic rings. PAHs are the perfect example of ubiquitous environmental pollutants [130]. Such biochar contains PAHs in a large quantity when used for land applications transported in soil. It also shows the bioavailability of PAHs when biochar is amended to other matrices. Hence the stability of biochar required more attention based on their properties [131]. The price of such biochar depends on the transportation or storage facilities and their pre-processing process [132].

Several ways of resource recovery have been developed and reviewed in the past years to improve the recovery of the materials or energy from the solid waste while reusing, reducing, and recycling [133], and biochar also play a wider role in resource recovery [134]. In addition to the inherent safety concerns, it also addresses exposing the external toxic chemicals during biochar modification as well as harmful antibiotic adsorbed biochar, and their subsequent lethal effects on organisms or on the entire ecosystem should not be disregarded. For example, graphene and other nanoparticles on the surface of biochar may encourage lethal effects on creatures via diverse mechanisms *e.g*., oxidative stress, cytotoxicity, and proteins inactivation [135]. Furthermore, microbes colonized in biochar pores may turn into violent species or antibiotic-resistant species, initiating ecological calamity upon application [136]. The re-mobilization of soil contaminants reported as biochar facilitates their transportation via biochar colloids [137]; however, their faded re-mobilization performances are reported with long-term aging. Researchers have raised a debate about its safe application [138]. Before, the land application of biochar, all safety concerns must be carefully monitored.

Feasibility in biochar costing may depend on biochar availability, which greatly depends on the usage of cheaper raw materials such as solid waste or agricultural, and forestry wastes, and cheaper production processes. To achieve the desirable well-carbonized biochar, it must be produced under controlled pyrolysis conditions and a consistent heating rate ensuring the right temperature range depending on the required surface property for specified applications. In addition, during the pyrolysis process, bio-oil and biogas are also produced, while the preparation or ongoing production of biochar, which is supportive for further energy recovery and offsetting production cost [139]. The distribution and transportation costs also affect its long-term development and maintenance benefits to the manufacturer [140]. Therefore, the production facility must be closeby to the biomass generation and distribution facilities. In addition to cost-effective production, optimized dosing of biochar is important for feasible antibiotic remediation. Moreover, it is important to determine a precise dosing rate as well as the correct particle size obtain maximum biochar reactivity and removal performance.

Antibiotics are among the refractory pollutants that accumulate in pharmaceutical wastewater; their concentration in wastewater treatment plants that treat antibiotic production wastewater has also remained high [141]. In the absence of effective controls, antibiotics in pharmaceutical wastewater may not only have direct impacts on environmental microbes but also could lead to an increase in antibiotic resistance among the environment’s microbes, which would pose a health risk to humans [142]. Therefore, we must utilize advanced treatment processes to deep remove the refractory pollutants, especially antibiotics, in pharmaceutical wastewater treatment plants. Considering the severity of the antibiotic pollution in the environment and the inability of adequate microbial activity with increasing concentration is a major challenge. Based on the progress achieved in biochar research, it is important to develop designer biochar for removing specifically each class of antibiotics which could be a promising, environmentally friendly, and cost-effective solution [26].

Biochar’s role is becoming pioneering to reduce the direct stress of antibiotics on microorganisms which helps directly and indirectly for antibiotic pollution removal [122,123]. Biochar has been recognized as a promising conductive mediator in the bioremediation system for improved organic degradation [122,123]. Especially engineered biochar with improved ability to increase the conductivity of mixed culture system to promote the bioremediation process is well covered using direct interspecies electron transfer [125]. Biochar application in microbial biodegradation system not only improved the desired product yield but also reinforced the microbial degradation of antibiotic pollutants via co-metabolism or electron exchange mechanism [122,123]. Future research endeavors can be focused in the following areas:

1. Further studies on catalysis potential determination of biochar (native and engineered) directly on antibiotic degradation as well as an indirect role in the reduction of direct stress on microbial performance are required. Such studies will be pioneering to design effective remediation methods for antibiotic polluted environments in the near future with designer biochar.
2. For engineering of biochar, researchers must focus on improving the biochar ability to activate peroxymonosulfate (PMS) and sodium percarbonate. They act as activators of redox reaction to efficiently exchange required electron for antibiotic biodegradation process.

## 8. Conclusions

Biochar-based antibiotic remediation is emerging as the most promising method due to its cost-effectiveness, and efficiency. As a result of different pyrolysis conditions, biochar produced at different temperatures has different interactions and removal efficiencies. There are different types of interactions depending on the biochar surface functionalities such as ion exchange, partitioning, π-π stacking, electrostatic attraction, H-bonding, etc. However, beyond their intrinsic ability to remediate antibiotic pollutants, important factors such as pH, temperature, biochar dosing, and initial pollutant concentration play an important role. Among them, pH was found to be the most important governing factor enabling biochar two- to three-fold more removal capacity than that of unoptimized pH removal. Physico-chemical and biological modification enhances native biochar properties significantly to have better removal efficiency for antibiotic pollutants, which was ranging 13–552 mg/g by dosing rate of 1–10 g/L. From the review analysis, biochar obtained from agri- and forestry biomass wastes at 500–700 pyrolysis temperature range was the best performing (274–552 mg/g), and the basis of adsorption involved mainly π-π electron donor––acceptor interaction, H-bonding, and electrostatic attractions. Based on the review analysis, it is important to get more insights into biochar kinetics studies for a multifactorial role in antibiotic removal mechanisms. Moreover, for the eventual disposition of various toxic antibiotic pollutants, it is important to degrade them effectively rather than remove them. Very limited studies have been carried out to establish the biochar-microbial role and their efficiency as compared to biochar alone. More studies must be carried out to compile the advantages of biochar’s role in improved microbial degradation for various antibiotics.

## Figures and Tables

**Table:** 

Tetracycline-TC	Sulfonamides -SNM	Erythromycin-ERY
Sulfamethoxazole-SMX	Sulfapyridine-SPY	Clarithromycin-CLA
Activated biochar -IA-BCs	Trimethoprim-TMP	Ampicillin-AMP
Ofloxacin-OFL	Sulfamethoxazole-SMX	Chlortetracycline-CTC
Ciprofloxacin-CIP	Tylosin-Tyl	Oxytetracycline -OTC
Norfloxacin -NOR	Levofloxacin-LEV	Doxycycline hydrochloride-DOX
Pefloxacin -PEF	Peroxymonosulfate (PMS)	Lignocellulosic biomass-LCB
